# Meta-analysis of the short-term effects of lamivudine treatment for severe chronic hepatitis B

**DOI:** 10.1186/1743-422X-10-134

**Published:** 2013-04-29

**Authors:** Lin Zhang, Chun-Qiu Hao, Jiang-Fu Liu, Meng Wang

**Affiliations:** 1 Department of Infectious Disease, Affiliated Sheng Jing Hospital of China Medical University, Sanhao Street of Heping District, Shenyang, Liaoning Province 110004, China; 2 Department of Infectious Diseases, Tangdu Hospital, Fourth Military Medical University, Xi'an, Shannxi Province 710038, China

**Keywords:** Lamivudine, Liver failure, Severe hepatitis, HBV, Meta-analysis

## Abstract

**Purpose:**

To evaluate the short-term effect of lamivudine (LMV) treatment for severe chronic hepatitis B.

**Method:**

Patient data related to the safety and efficacy of using lamivudine (LMV) to treat hepatitis B virus (HBV)-induced liver failure or severe hepatitis were acquired from previous literature. These studies were retrieved from PubMed, Ovid, SpringerLink, Biosis Previews, Academic Search Premier, ProQuest Medical Library, Cochrane Library, China National Knowledge Infrastructure Full-text Database, VIP Chinese Scientific Journal Database, and Chinese Biomedicine. Relative risk and weighted mean difference were used to measure the effects. The major predictors observed included total bilirubin (TBIL), prothrombin activity (PTA), survival rate, and HBV-DNA negative change rate. Groups were further divided according to the clinical course and disease staging.

**Results:**

A total of 242 studies were retrieved from the databases. At weeks 4, 8, and 12 of the treatment course, the survival rates and PTA of the test group were distinctively higher than those of the control group. However, TBIL concentrations in the test group were lower than the control group. The HBV-DNA negative change rate was distinctively higher throughout the 12 weeks of LMV treatment. For patients who started LMV treatment in the middle stage, the mortality rate of the test group was lower. For patients who started LMV treatment during the advanced stage, no significant difference was observed between the test and control groups.

**Conclusion:**

LMV decreased HBV-DNA levels in the serum, improved liver function in patients, and enhanced survival rate during the early and medium stages of severe chronic hepatitis B.

## Introduction

Severe hepatitis is a syndrome characterized by liver failure due to the death or severe degeneration of a large number of liver cells. This severe, progressive, and complex syndrome frequently results in multiple organ failure. Severe hepatitis has a mortality rate of over 70% [1-3]. Various hepatitis virus types can lead to severe hepatitis. In China, hepatitis B virus (HBV) infection is the leading cause of severe hepatitis [4]. One of the important mechanisms of severe hepatitis is the high level of HBV replication and protein antigen expression on target cell surfaces, which often leads to cytotoxic T lymphocyte (CTL)-mediated immune response. This mechanism causes death to large numbers of liver cells [5-7]. Researchers have hypothesized that liver cell damage could be controlled by inhibiting HBV replication to inhibit immune responses [8]. No safe and effective drug for severe hepatitis B existed before nucleoside analogs were used as alternative options. Current treatments for severe hepatitis include supportive and symptomatic treatment-based comprehensive treatment, artificial liver support systematic treatment, and liver transplant [9]. With the increasing development and clinical use of nucleoside analog drugs, an increasing number of studies have also been conducted. However, these studies have not reached a consensus. Several studies have indicated that these drugs help lower the mortality rate and improve liver function in patients. However, other studies also suggest that nucleoside analog drugs have been ineffective. In addition, other studies have reported that the increase of liver failure cases is attributed to lamivudine (LMV) use. Nevertheless, the adverse effect of LMV on patients with hepatic insufficiency is still unclear. Therefore, HBV-related liver failure cannot be listed as the indication for LMV.

In “AASLD Position Paper: The Management of Acute Hepatitis,” “Guidelines for the Diagnosis and Treatment of Liver Failure,” and “Chronic and Acute Liver Failure Consensus” released by the Asia-Pacific Liver Disease Association, nucleoside analog drugs were recommended as antivirus treatment. However, the level of evidence is only Grade 3 (experience and comments by specialists or authority). In the present study, studies published before December 2010 were reviewed to evaluate the outcome of the LMV treatment for HBV-related liver failure using evidence-based perspectives. A meta-analysis was conducted to evaluate the efficacy of LMV in treating severe chronic hepatitis B.

## Materials and methods

### Literature search strategy

The data base for our research include PubMed, Ovid, SpringerLink, Biosis Previews, Academic Search Premier, ProQuest Medical Library, Cochrane Library, China National Knowledge Infrastructure Full-text Database, VIP Chinese Scientific Journal Database, and Chinese Biomedicine. The timeframe for literature search is from the establishment dates of these databases until December 2010. The subject term included “hepatic failure” OR “liver failure” OR “severe hepatitis B” OR “hepatitis B virus” OR “HBV”, AND “lamivudine” AND “RCT” OR “CCT” OR “cohort study”. And absence of any language restrictions.

### Inclusion and exclusion criteria

The following were the inclusion criteria:

(1) Research object: severe chronic hepatitis B, consistency of diagnosis and staging with the diagnosis criteria proposed by “Prevention and treatment scheme for virus-related hepatitis” [10], released in 2000, or “Guidelines for the diagnosis and treatment of liver failure,” released in 2006;

(2) HBV-DNA>10^3^ copies/ml;

(3) Interventional measure: routine comprehensive treatment in the control group; LMV (100 mg/d) combined with routine comprehensive treatment in the test group;

(4) The neutrality and comparability of the two groups studied in terms of age, gender, and other biological or chemical predictors; and

(5) Published status and full text availability of the studies considered.

The following were the exclusion criteria:

(1) Reports involving concurrent infection with hepatitis A, C, D, and E virus, Epstein-Barr virus, cytomegalovirus, HIV, and others;

(2) Reports involving concurrence with drug-induced liver injury, auto-immune liver disease, alcoholic liver disease, and inherited metabolic disease, among others.

(3) Reports involving concurrence with malignant tumors and severe blood anomalies.

(4) Reports covering only adverse effects or descriptive studies;

(5) Non-convertible or unusable data in literature;

(6) Reports involving comparisons or use of LMV with Chinese medicine; and

(7) Reports involving concurrence with plasma exchange treatment.

### Indicators of therapeutic efficacy

Survival, total bilirubin (TBIL), prothrombin activity (PTA), and HBV-DNA negative change rates were compared between the test and control groups at weeks 4, 8, and 12. These rates were considered indicators for evaluating LMV efficacy. The effects of starting LMV treatment at different time points during the clinical course of liver failure were also observed.

### Quality evaluation and information collection

At least two evaluators were involved in the selection of studies to conduct quality evaluations and information collection independently before exchanging evaluation results with one another. In cases of disagreement, a third evaluator settled the dispute. The quality evaluation for RCT was conducted according to the Jadad measuring scale [11], whereas that for CCT was conducted according to the Stroup criteria [12].

The information collected included basic information on the studies, sample sizes, intervention characteristics (intervention measures, dosage, course, follow-up, etc.), receiver characteristics (gender, age, TBIL, PTA, virus infection, complication, etc.), and results.

### Statistical analysis

Revman 4.2 was used for statistical analysis. Among the indicators compared, relative risk (RR) and 95% confidence interval (95% CI) were used to compare the mortality, survival, and HBV-DNA negative change rates. Weighted mean difference (WMD) and 95% CI were used to compare TBIL and PTA. Based on the suggestion of the Journal of American Medical Association (JAMA) [13], a homogeneity test was used to analyze the heterogeneity of the data collected. A fixed model was used in meta-analysis. A *P* value greater than 0.05 indicated no statistical heterogeneity, whereas a *P* value less than 0.05 indicated statistical heterogeneity. Subgroup and sensitivity analyses were used to exclude the suspected cause for clinical and statistical heterogeneity. If heterogeneity still existed, the random effect model was used in meta-analysis. A funnel chart was used to detect possible publication biases. Results were further tested by sensitivity analysis.

## Results

### Inclusion of basic information on studies retrieved

**Figure 1:**
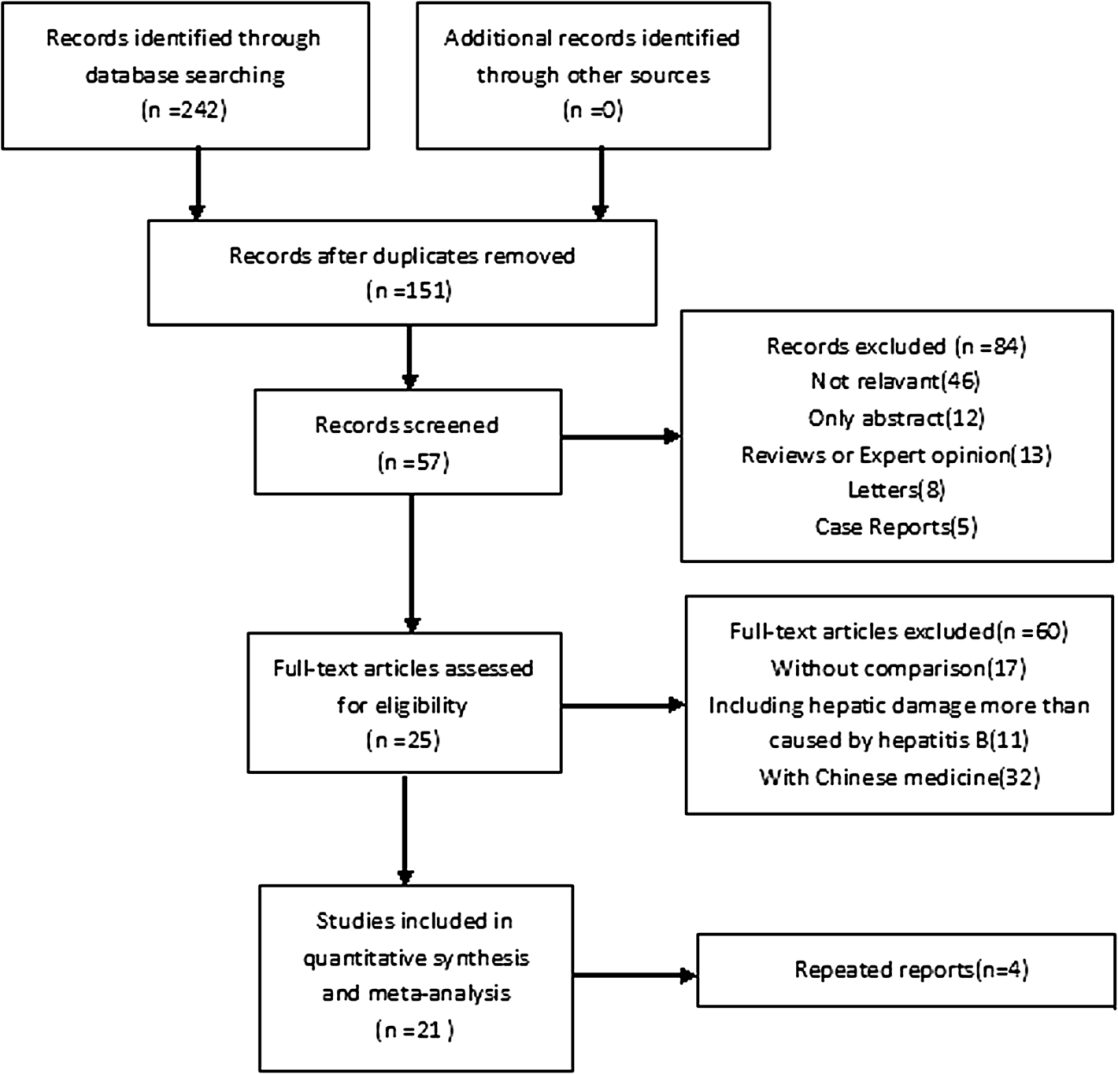
Flow diagramme.

A total of 242 relevant studies (97 in English, 145 in Chinese) were retrieved from the databases, and 21 were ultimately included after literature screening. Of the 21 studies included, 13 were RCT and 8 were cohort studies. A total of 780 cases were included in the test group, whereas 768 cases were included in the control group. The process of selecting comparative studies included in our meta-analysis was shown in Figure 1. The characteristics and quality assessments of included studies are shown in Table 1.

**Table 1:** **Characteristics of studies included in the meta**-**analysis**

**Study**	**Year**	**Country**	**Group**	***n***	**M/F**	**Age(yr)(mean ±SD**)
Sun LJ et al. [14]	2009	China	Test	130	104/26	N/A
			Cont.	130	104/26	N/A
Cui YL et al. [15]	2010	China	Test	34	3/31	39.35 ± 10.61
			Cont.	37	6/31	41.03 ± 11.48
Mi Lj et al. [16]	2009	China	Test	30	30/712	38.4 ± 2.5
			Cont.	28	24/3	38.3 ± 2.4
Zhang P [17]	2008	China	Test	48	38/10	47 ± 15.8
			Cont.	44	36/8	48 ± 16.2
Han YH et al. [18]	2007	China	Test	58	36/22	N/A
			Cont.	48	29/19	N/A
Yang DH et al. [19]	2004	China	Test	30	N/A	N/A
			Cont.	41	N/A	N/A
He Y et al. [20]	2005	China	Test	13	11/2	38.2
			Cont.	24	21/3	37.8
Liao JH et al. [21]	2004	China	Test	36	29/7	39.5 ± 17.6
			Cont.	40	32/8	38.6 ± 16.8
Yuan J et al. [22]	2001	China	Test	20	17/3	35.1 ± 11.6
			Cont.	20	20/0	34.8 ± 8.5
Zhang LQ et al. [23]	2006	China	Test	29	24/5	N/A
			Cont.	20	18/2	N/A
Zhan GQ et al. [24]	2006	China	Test	62	N/A	N/A
			Cont.	36	N/A	N/A
Cao L et al.[25]	2007	China	Test	45	N/A	N/A
			Cont.	46	N/A	N/A
Zhu GL et al. [26]	2002	China	Test	31	25/6	N/A
			Cont.	31	23/8	N/A
Zhou BX et al. [27]	2011	China	Test	21	16/5	37.2
			Cont.	21	15/6	38.1
Guo JC et al. [28]	2002	China	Test	24	24/0	38 ± 17.1
			Cont.	24	24/0	39 ± 18.4
Zhong YB et al. [29]	2007	China	Test	24	20/4	29.2 ± 16.8
			Cont.	35	29/6	33.8 ± 18.7
Zhou XX et al. [30]	2007	China	Test	17	14/3	N/A
			Cont.	18	15/3	N/A
Ding FY et al. [31]	2003	China	Test	38	31/7	N/A
			Cont.	38	33/5	N/A
Pan JZ et al. [32]	2007	China	Test	40	N/A	N/A
			Cont.	42	N/A	N/A
Wang Y et al. [33]	2003	China	Test	30	N/A	N/A
			Cont.	30	N/A	N/A
Qin SJ et al. [34]	2003	China	Test	20	N/A	N/A
			Cont.	15	N/A	N/A

### Literature quality

**Figure 2:**
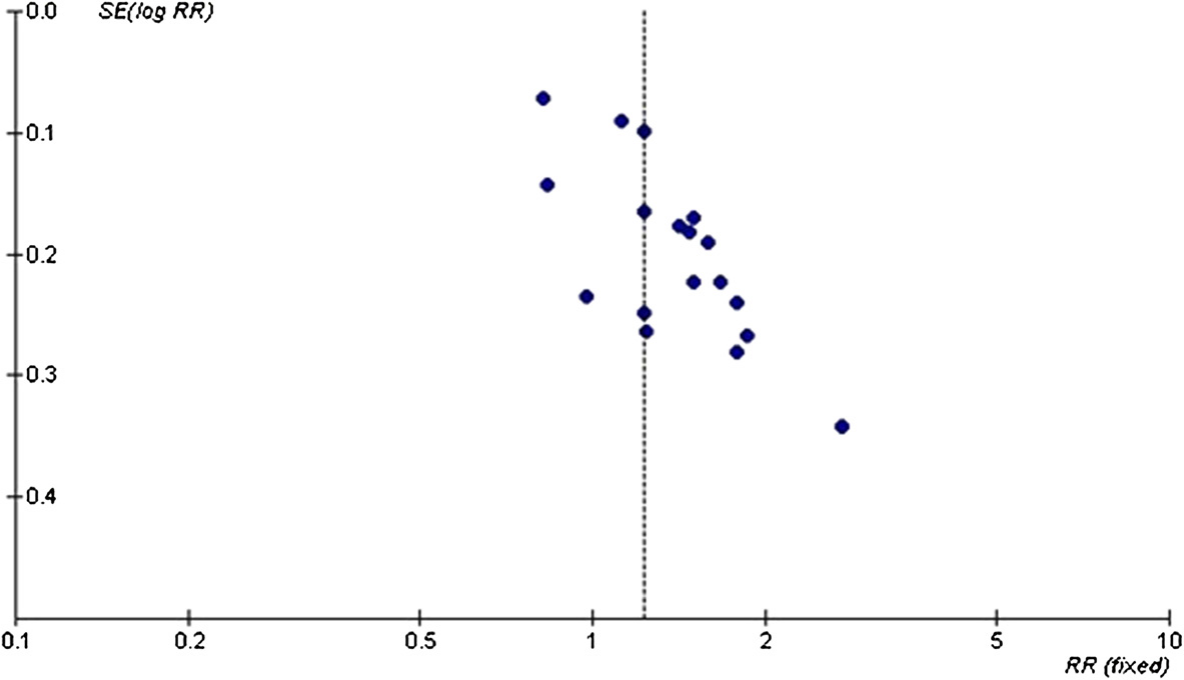
Funnel plot for publication bias analysis.

As shown in Figure 2, the funnel plot, which indicates the effect size measures of the studies included, was symmetrically scattered on both sides of the real value. Thus, publication bias was unlikely. A sensitivity test was performed to test the reliability of meta-analysis. Studies with treatments that varied largely from the real value were excluded. After the sensitivity test, the remaining studies were subjected to another test. No indication of publication bias was found.

### Effect of LMV treatment on the survival rate

**Figure 3:**
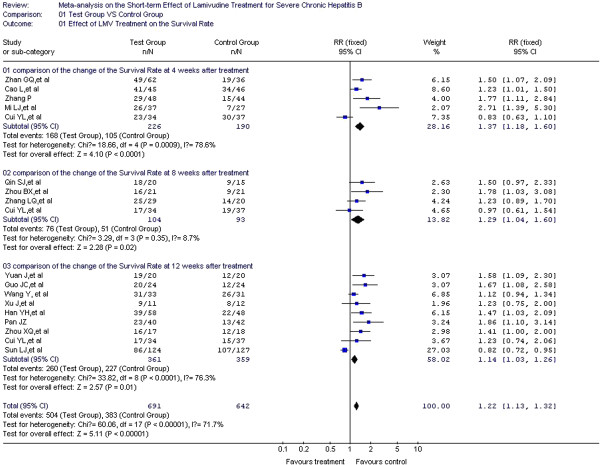
Effect of LMV treatment on the survival rate.

Survival rate was considered predictive of prognosis in 16 of the studies included [14-19,22-25,27,28,30,32-34] with 638 cases in the test group and 600 cases in the control group. In the heterogeneity test, *χ*^2^=60.06, df=17, and *P*<0.00001, suggesting a certain degree of heterogeneity among the included studies. Therefore, the random effect model was used to combine the effect measures using RR as indicator (RR=1.22), and 95% CI was (1.13, 1.32). In the forest plot, the diamond was completely on the right side of the vertical line. In the test for the overall effect, Z=5.11 and *P*<0.00001, suggesting that LMV treatment in patients with severe chronic hepatitis B improved survival rate. A significant statistical difference between the test and control groups was observed. Heterogeneity in the subgroup analysis was confirmed during the treatment course (weeks 4, 8, and 12). Therefore, the random effect model was used for the subgroup analyses. The RRs were 1.37, 1.29, and 1.24, respectively, and the 95% CIs were (1.18, 1.60), (1.04, 1.6), and (1.03, 1.26), respectively. In the test for the overall effect, *P* < 0.05, indicating a significant statistical difference. Thus, LMV improves the survival rate of patients at weeks 4, 8, and 12 during the treatment course (Figure 3).

### TBIL comparison between test and control groups

**Figure 4:**
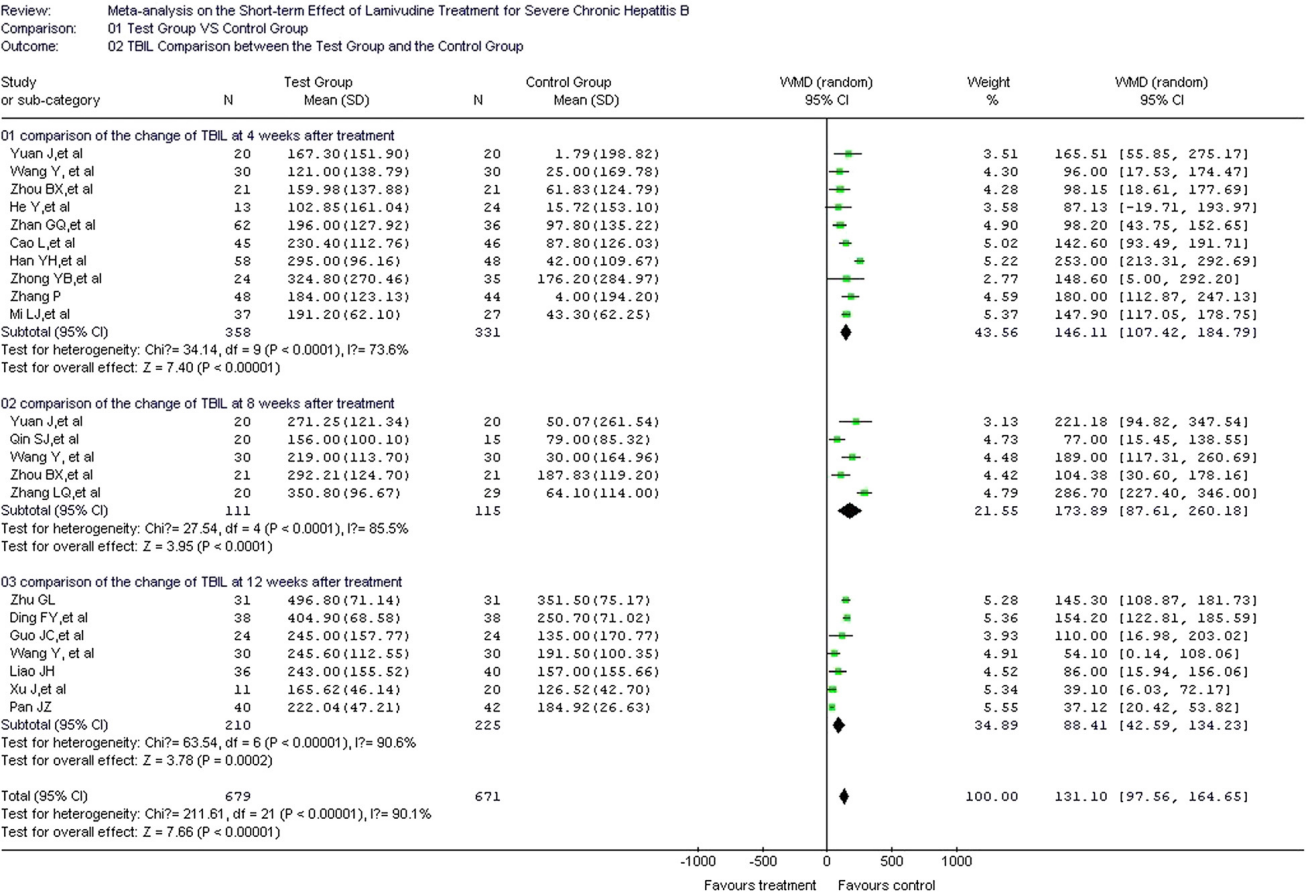
Effect of LMV treatment on TBIL.

TBIL was considered predictive for prognosis in 18 of the included studies [16-29,31-34], with 599 cases in the test group and 583 cases in the control group. In the heterogeneity test, *χ*^2^=211.61, df=21, and *P*<0.00001, suggesting a certain size of heterogeneity among the included studies. Therefore, the random effect model was used to combine the effect measure using WMD as the indicator (WMD=131.1), and 95% CI was (97.56, 164.65). In the forest plot, the diamond was completely on the right side of the vertical line. In the test for the overall effect, Z=7.66, and *P*<0.00001. Thus, LMV decreases the TBIL level in patients. A significant statistical difference was observed between the test and control groups. Heterogeneity was confirmed in the treatment course subgroup analysis for weeks 4, 8, and 12. Therefore, the random effect model was used for the subgroup analyses. The overall WMDs were 146.11, 173.89, and 88.41, respectively. The 95% CI were (107.42, 184.79), (87.61, 260.18), and (42.59, 134.23), respectively. In the test for the overall effect, *P* < 0.05, indicating a significant difference between the test and control groups. LMV treatment thus decreases the TBIL level at weeks 4, 8, and 12 during the treatment course (Figure 4).

### PTA comparison between test and control groups

**Figure 5:**
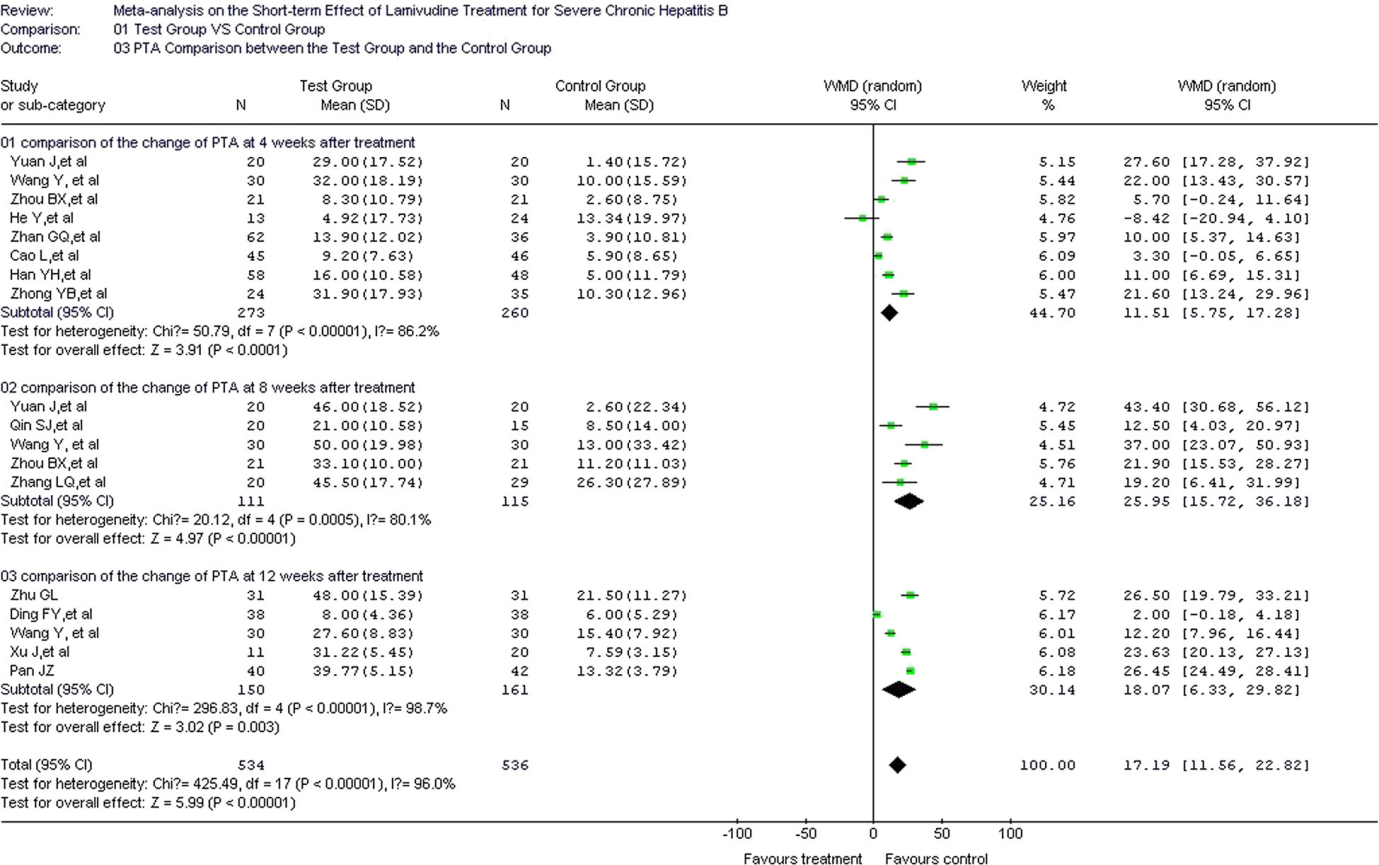
Effect of LMV treatment on TBIL.

PTA was considered the predictor for prognosis in 14 of the included studies [18-20,22-27,29,31-34], with 461 cases in the test group and 447 cases in the control group. In the heterogeneity test, *χ*^2^=425.49, df=13, and *P*<0.00001, which suggest a certain size of heterogeneity among the studies included. Therefore, the random effect model was used to combine the effect measure, WMD. WMD=17.19, and 95% CI was (11.56, 22.82). In the forest plot, the diamond was completely on the right side of the vertical line. In the test for the overall effect, Z=5.99, and *P*<0.00001. LMV treatment thus increases PTA levels in patients and improves coagulation function. A significant statistical difference was observed between the test and control groups. Heterogeneity was confirmed in the treatment course subgroup analysis for weeks 4, 8, and 12. Therefore, the random effect model was used for the subgroup analyses. The overall WMD were 11.51, 25.95, and 18.07, respectively, and overall 95% CI were (5.75, 17.28), (15.72, 36.18), and (6.33, 29.82), respectively. In the test for the overall effect, *P* < 0.5, indicating a significant difference between the test and control groups. Thus, LMV treatment increases the PTA level at weeks 4, 8, and 12 during the treatment course (Figure 5).

### Comparison of HBV-DNA negative change rate between test group and control group

**Figure 6:**
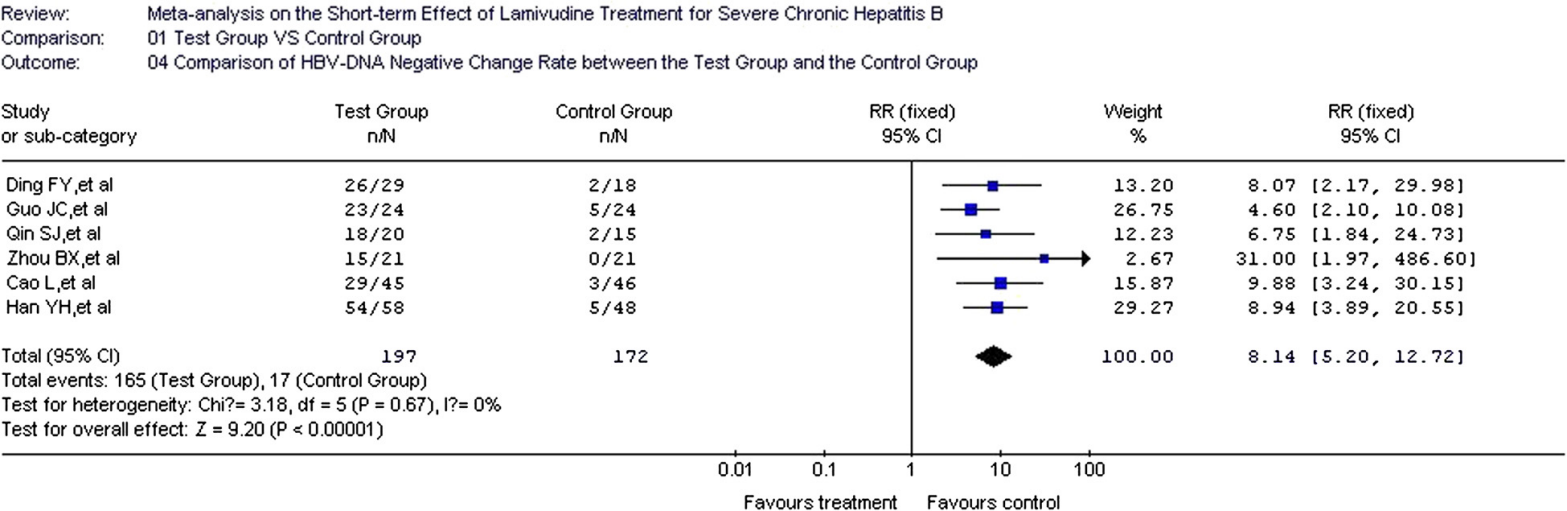
**Effect of LMV treatment on HBV**-**DNA negative change rate.**

The HBV-DNA negative change rate was considered the predictor for prognosis in six of the included studies [18,25,27,28,31,34], with 206 cases in the test group and 192 cases in the control group. In the heterogeneity test, *χ*^2^=3.18, df=5, and *P*=0.67>0.05, which suggest no substantial heterogeneity among the six studies. Therefore, the fixed effect model was used to combine the effect measure, RR. RR=8.14, and 95% CI was (5.2, 12.72). In the forest plot, the diamond was completely on the right side of the vertical line. In the test for the overall effect, Z=9.2, and *P*<0.00001. Thus, LMV treatment significantly inhibits HBV-DNA change. The negative change rate in the test group was 14 times higher than that in the control group, which also signifies a statistically significant difference (Figure 6).

### Effect of LMV treatment at different stages of liver failure on the prognosis

Four of the included studies [17,18,29,32] were divided into three stages, namely, early, medium, and advanced. This classification was done according to “Diagnosis and Prevention Scheme for Viral Hepatitis.” Mortality rate was used as the indicator for prognosis.

Fifty-nine cases were included in the early stage test group and 54 in the control group. In the heterogeneity test, *χ*^2^=0.96, df=3, and *P*=0.81>0.05, which suggests no substantial heterogeneity among the studies included. Therefore, the fixed effect model was used to combine the effect measure, RR. RR=0.21, 95% CI was (0.09, 0.51). In the forest plot, the diamond was completely on the left side of the vertical line. In the test for the overall effect, Z=3.46, and *P*=0.0005. A significant difference was observed between the test and control groups. Thus, LMV treatment during the early stage decreases the mortality rate.

Seventy cases were included in the medium stage test group and 72 in the control group. In the heterogeneity test, *χ*^2^=2.0, df=3, and *P*=0.56>0.05, which suggests no substantial heterogeneity among the studies included. Therefore, the fixed effect model was used to combine the effect measure, RR. RR=0.43, and 95% CI was (0.28, 0.66). In the forest plot, the diamond was completely on the left side of the vertical line. In the test for the overall effect, Z=3.83, and *P*=0.0001. A significant difference was observed between the test and control groups. Thus, LMV treatment in the early stage decreases the mortality rate.

**Figure 7:**
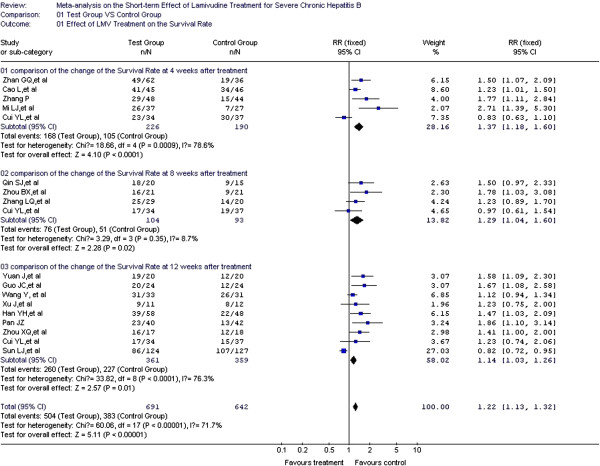
Effect of LMV treatment on mortality rate of patients in different stages.

Forty-one cases were included in the advanced stage test group and 35 in the control group. In the heterogeneity test, *χ*^2^=0.11, df=1, and *P*=0.73>0.05, which suggests no substantial heterogeneity among the studies included. Therefore, the fixed effect model was used to combine the effect measure, RR. RR_c_=0.98, and 95% CI was (0.79, 1.22). In the forest plot, the diamond overlapped with the no-effect vertical line. In the test for the overall effect, Z=0.17, and *P*=0.86, suggesting no significant difference between the test and control groups in the advanced stage (Figure 7).

### Adverse effect

No adverse effect was identified in the studies included.

## Discussion

The mechanism for severe hepatitis is rather complicated and remains unclear. Current understanding on HBV-related liver failure/severe hepatitis holds that liver tissues undergo irreparable damage from immunologic injury, ischemia, hypoxia, and endotoxemia [35]. Among these conditions, HBV replication has a primary and dominant function in the onset of these diseases [36-38]. HBV replication leads to the hyperfunction of immune responses, especially the CTL-mediated cellular immune response. A retrospective study on the cause and outcome of chronic and acute liver failure conducted by Zhao et al. [39] suggests that HBV replication and mutation primarily cause severe hepatitis B. Therefore, a reasonable solution for treating severe hepatitis B is to reduce the total number of viruses within the body and relieve immune hyperactivity using antivirus drugs.

Several studies claim LMV treatment during the early stage and before the bilirubin level exceeds 20 mg/dl can improve the prognosis and reduce mortality rate. However, untimely LMV treatment will lose its effectiveness [40-42]. Thus, the key to reducing the mortality rate of severe chronic hepatitis B is early treatment. Antivirus treatment is especially crucial for patients confirmed for HBV replication-associated liver damage to protect the unaffected liver cells. A retrospective matched cohort study by Sun et al. [14] reviewed 130 cases of severe chronic hepatitis B patients receiving LMV treatment. All patients were observed for 3 months of follow-up. The test group was found to have a higher survival rate than the control group (*P*=0.0021), and patients with a larger number of viruses had a higher mortality rate. The Cox proportional hazards model was used to evaluate predictors relating to patient prognosis. Patients with a model for end-stage liver disease (MELD) score between 20 and 30 showed a significantly lower mortality rate if treated with LMV during the early stage. However, LMV treatment did not improve outcomes in patients with MELD scores over 30. In the report of Yu et al. [43], the significant drop in HBV-DNA is an important predictor for the prognosis of severe chronic hepatitis B patients with MELD scores between 30 and 40. LMV is well tolerated in all patients, suggesting LMV is a safe and effective drug for treating severe chronic hepatitis B.

However, several reports claim that antivirus treatment, mostly LMV treatment, does not have good clinical effects on patients with severe chronic hepatitis. B. Yuen et al. [44] claim that even early LMV treatment cannot improve the prognosis of patients with severe chronic hepatitis B. In a study done by Tsubata et al. [45], 25 patients with severe chronic hepatitis B received regular treatment combined with LMV. Another 25 patients were placed in the control group. Six patients in the test group (24%) and seven in the control group (28%) almost immediately underwent liver failure. Among those patients, three from the test group and two from the control group survived. However, this gap cannot represent a statistical difference in supporting the efficacy of LMV in inhibiting the progress of severe chronic hepatitis B. In the research by Cui et al. [15], 104 patients with severe chronic hepatitis B were divided into three groups, namely, the LMV, entecavir (ETV), and routine groups. During 3 months of follow-up observation, the survival rates for the three groups were 48.49%, 50%, and 40.54%, respectively. No significant difference was observed in the survival rate (*P*=0.72), in liver function and kidney function, and MELD score. However, five patients from the routine group had a relapse. Even though LMV and ETV are not effective in improving the prognosis of patients in the short-run, both can help reduce the relapse rate for severe chronic hepatitis B. Thus, patients may benefit from long-term effects. Researchers also claim that the prolonged use of LMV increases the risk of drug resistance. Once tyrosine-methionine-aspartate-aspartate mutation occurs, the outcome achieved is endangered and even exacerbates patient conditions. In the research by Wong et al. [46], 45 patients with severe chronic hepatitis B and positive hepatitis B ‘e’ antigen (HBeAg) received LMV treatment. The test group showed a significantly higher HBeAg seroconversion rate than the control group (73% vs. 52%). However, 33% of patients in the LMV group exhibited drug resistance and relapse in 5 years, and 73% patients suffered from exacerbated disease conditions.

The effect of LMV on severe chronic hepatitis B induced by HBV is still unconfirmed and is a topic of disagreement among researchers. LMV is a type of pyrimidine nucleoside analog that can inhibit the activity of DNA polymerase and reverse transcriptase. This inhibition decreases HBV replication, thereby reducing the number of viruses in the liver and blood, decreasing the target antigen expression on the surface of liver cells, and reducing CTL attacks on infected liver cells. Thus, both the primary and sustaining factors can be effectively controlled. In the present research, CCTs conducted before December 2010 that aimed to compare the effects of LMV and routine treatments on severe chronic hepatitis B were collected. Through meta-analysis, LMV was found to inhibit HBV replication. TBIL, PTA, and survival rates all showed significant improvements. Thus, inhibiting HBV replication can inhibit HBV replication in infected liver cells, thereby reducing the risk of new infections. Thus, inhibiting HBV replication relieves the inflammatory reaction in the liver, boosts liver cell function recovery, and improves the prognosis of patients.

LMV treatment during the early and medium stages can decrease the mortality rate of patients with severe chronic hepatitis B. However, for the advanced stage, no significant difference was found between the test and control groups. Antivirus treatment can prolong the lives of patients, which provides valuable time for liver cell reproduction. However, during the advanced stage, patients usually suffer from significantly worse liver cell death and are also more likely to suffer from complications, accelerating patient death. Patients can benefit from antivirus treatment during the early and medium stages, but a liver transplant is a more reasonable choice for patients in the advanced stage.

Currently, the four types of nucleoside drugs available in the Chinese market include LMV, telbivudine, ETV, and adefovir dipivoxil. LMV, telbivudine, and ETV have rapid curative effects with powerful antiviral capacity, whereas adefovir dipivoxil has a slow effect and results in the adverse reaction of increased serum creatinine. When this reaction occurs, adefovir dipivoxil is not applicable in treating hepatic failure. Among the former three types of nucleoside drugs, ETV can lead to lactic acidosis in patients with severe hepatitis and cirrhosis. Telbivudine can result in increased creatine kinase. Therefore, neither ETV nor telbivudine are less than LMV. Although LMV is associated with a high drug resistance rate during long-term treatment, this rate does not influence its application in treating hepatic failure. Consequent problems such as drug resistance can be managed by follow-up monitoring.

Severe hepatitis occurs as a series of pathological and physiological processes initiated by the primary immunologic injury of liver cells and prompted by massive liver cell death. Liver cell death is caused by the endotoxemia-induced release of cytokines and inflammatory mediators [47-50]. Several studies claim that severe chronic hepatitis B can progress even though HBV-DNA levels have been controlled within an undetectable range, causing patientdeath. Thus, viral infection is not the only factor for the progression of severe chronic hepatitis B. Other than liver transplant, we believe that another reasonable treatment is to give patients an early antivirus treatment based on proactive primary care (rest, hepatoprotective treatment, symptomatic treatment, artificial liver support, etc.).

Meta-analysis is as a novel way of literature review. Meta-analysis can comprehensively evaluate and quantitatively analyze multiple research results from a systematic and objective perspective, thereby improving the efficiency of statistical analysis and tests. Although a large number of studies were included in the present research, analyses were also conducted to evaluate the publication bias of such studies. The present research is not different from others in terms of its subjection to publication bias due to the possibility of unpublished studies with negative results. Furthermore, the objectiveness and accuracy of meta-analysis largely depend on a pool of high-quality literature. Due to the limitation of disease categories and ethical reasons, large-scale, multi-centered RCTs are impractical for studies like the present paper. Several flaws exist in the literature inclusion method. These limitations have all affected the stability of research results. Therefore, the aforementioned conclusions still await further research with increased high-quality random controlled research literature included.

## Competing interests

Authors declare that they have no competing interests.

## Authors’ contributions

Study conception and design: LZ and C-QH. Data acquisition and analysis: J-FL. Drafting of manuscript: MW. Critical revision: LZ. All authors read and approved the final manuscript.
